# Long-Term Toxicity of ^213^Bi-Labelled BSA in Mice

**DOI:** 10.1371/journal.pone.0151330

**Published:** 2016-03-16

**Authors:** Laëtitia Dorso, Edith Bigot-Corbel, Jérôme Abadie, Maya Diab, Sébastien Gouard, Frank Bruchertseifer, Alfred Morgenstern, Catherine Maurel, Michel Chérel, François Davodeau

**Affiliations:** 1 Nuclear Oncology, Nantes-Angers Cancer Research Center (CRCNA) UMR 892 Inserm, Nantes, France; 2 University of Nantes, Nantes, France; 3 CNRS, UMR 6299, Nantes, France; 4 Biochemistry Department, Laënnec Hospital, Nantes, France; 5 L'UNAM University, Oniris, AMaROC Unit, Nantes, France; 6 European Commission, Joint Research Centre, Institute for Transuranium Elements, Karlsruhe, Germany; 7 Nuclear Medicine, ICO institut de Cancerologie de l'Ouest - Centre René Gauducheau, Nantes, France; 8 Nuclear Medicine, University Hospital, Nantes, France; Brandeis University, UNITED STATES

## Abstract

**Background:**

Short-term toxicological evaluations of alpha-radioimmunotherapy have been reported in preclinical assays, particularly using bismuth-213 (^213^Bi). Toxicity is greatly influenced not only by the pharmacokinetics and binding specificity of the vector but also by non-specific irradiation due to the circulating radiopharmaceutical in the blood. To assess this, an acute and chronic toxicity study was carried out in mice injected with ^213^Bi-labelled Bovine Serum Albumin (^213^Bi-BSA) as an example of a long-term circulating vector.

**Method:**

Biodistribution of ^213^Bi-BSA and ^125^I-BSA were compared in order to evaluate ^213^Bi uptake by healthy organs. The doses to organs for injected ^213^Bi-BSA were calculated. Groups of nude mice were injected with 3.7, 7.4 and 11.1 MBq of ^213^Bi-BSA and monitored for 385 days. Plasma parameters, including alanine aminotransferase (ALT), aspartate aminotransferase (AST), blood urea nitrogen (BUN) and creatinine, were measured and blood cell counts (white blood cells, platelets and red blood cells) were performed. Mouse organs were examined histologically at different time points.

**Results:**

Haematological toxicity was transient and non-limiting for all evaluated injected activities. At the highest injected activity (11.1 MBq), mice died from liver and kidney failure (median survival of 189 days). This liver toxicity was identified by an increase in both ALT and AST and by histological examination. Mice injected with 7.4 MBq of ^213^Bi-BSA (median survival of 324 days) had an increase in plasma BUN and creatinine due to impaired kidney function, confirmed by histological examination. Injection of 3.7 MBq of ^213^Bi-BSA was safe, with no plasma enzyme modifications or histological abnormalities.

**Conclusion:**

Haematological toxicity was not limiting in this study. Liver failure was observed at the highest injected activity (11.1 MBq), consistent with liver damage observed in human clinical trials. Intermediate injected activity (7.4 MBq) should be used with caution because of the risk of long-term toxicity to kidneys.

## Introduction

The high Linear Energy Transfer (LET) and the short path of alpha particles (a few tens of μm) enable tumour cells to be destroyed by fewer than ten alpha tracks per cell [1]. These properties are suited to targeting small clusters of cells, isolated cells in haematological pathologies, or for the treatment of micro metastasis in consolidation treatment. Targeted radionuclide therapy (TRNT) with alpha particles is thus complementary to TRNT with beta particles, whose millimetric path is better suited to bulky tumour treatment.

Contrary to external beam irradiation, which delivers well-known homogenous irradiation doses to a defined organ volume, the doses delivered to healthy organs during alpha or beta TRNT are much more difficult to estimate. Therefore, establishing the relationship between the absorbed dose and toxicity remains a challenge for medical dosimetry [2]. For particles with a short path length, the energy deposition within an organ is not homogenous and depends on the binding sites of the radiolabelled vector within tissue ultrastructures [1]. TRNT may induce kidney failure, which is isotope and vector-dependent at equivalent doses to organs [3]. Thus, the toxic effect of this irradiation depends firstly on the biodistribution of the radiolabelled vector, and secondly on the histological organ structure. For example, in the case of the liver, hepatocytes, whose cell diameter is in the range of 40 μm, are extensively irradiated by the radiolabelled vector circulating in the blood capillaries bordering them. The resulting crossfire effect leads to irradiation of the whole liver volume even without the specific uptake of the radiolabelled vector. Thus, in TRNT, the systemic injection of a radiopharmaceutical corresponds to a total body irradiation with variable doses and dose rates to each organ. There are few data enabling an evaluation of the radiation effect on quiescent cells, probably because of the inherent difficulties of working with these cells in tissue culture. The ionising radiation effect on healthy organs needs to be assessed by means of an overall monitoring of organ function and confirmed by histological examination of irradiated tissues.

Numerous ^213^Bi toxicity studies have been performed in the course of preclinical RIT assays [4–9]. Most of them were carried out during short-term studies on tumour-bearing mice and did not provide information on the long-term effects of a systemic irradiation with ^213^Bi [10, 11]. However, injected activities that effectively reduce tumour sizes and show no acute toxicity to healthy organs could result in long-term tissue damage and organ failure [5].

Toxicity is closely related to the pharmacokinetics of the vector used for TRNT. Given the short half-life of bismuth-213 (^213^Bi), the use of small-size vectors, such as radiolabelled peptides with fast pharmacokinetics, pre-targeted radioimmunotherapy (RIT) approaches, or antibody fragments like Fab’, enables high radiation doses to be delivered to the tumour within the time of ^213^Bi decay [6]. The rapid clearance of these kinds of vector from the blood limits their myelotoxicity. However, their elimination via the kidneys raises the possibility of renal toxicity, which appears in mice more than 10 weeks after treatment with infra-therapeutic doses [12].

Conversely, the use of larger vectors, like antibodies or F(ab')_2_ antibody fragments, to target tumours rapidly accessible to an effective blood supply limits kidney toxicity at the cost of prolonged irradiation of haematopoietic bone marrow and healthy organs due to the serum stability of the vectors and their low extravasation rate [11]. With such ^213^Bi-radiolabelled macromolecules, healthy organs are substantially irradiated from the blood content. Moreover, free bismuth also contributes to the global mean dose to organs due to dechelation from the vector that occurs over time. The elimination of bismuth is known to involve urinary and biliary excretion but the respective contributions of these two excretion routes is dependent on the bismuth formulation and the mode of administration. Dechelation of bismuth for macromolecules like soluble proteins, antibodies or Fab’2 antibody fragments is largely determined by the strength of the interaction between bismuth and the chelating agent used for radiolabelling.

This study thus aims to assess the long-term tissue damage in a preclinical setting by using ^213^Bi radiolabelled BSA as an example of a non-specific macromolecule vector. In fact, BSA has a slow diffusion rate across the vascular endothelium during the time of ^213^Bi decay [13]. Its size (66 kDa and 7 nm in diameter) is above the physiological upper limit of pore size in the walls of non-fenestrated blood capillaries [14]. Because BSA is larger than the limit of renal filtration, like F(ab')_2_ and IgG currently used in RIT, it is contained within the plasma volume. Therefore, using ^213^Bi-labelled BSA (^213^Bi-BSA) it is possible to evaluate the toxicity inherent in radioimmunotherapy with macromolecules due to either irradiation from the blood or the release of free bismuth following dechelation of ^213^Bi from the chelating agent CHX-A”-DTPA, currently used to radiolabel macromolecule vectors. ^213^Bi-BSA was injected intravenously into healthy nude mice, at increasing activities. Mice were monitored for 385 days after injection. Blood-cell counts, measurement of blood biochemical parameters and the histopathological examination of organs were performed during this period in order to understand better the acute and chronic alpha-emitter-induced radiotoxicity.

## Materials and Methods

### Animals

NMRI-nu (nu/nu) female mice, 7 to 8 weeks old and weighing between 28.4 and 34.8 g, were purchased from Janvier^®^, Le Genest St Isle, France. Mice were housed under standard conditions (standard diet and water *ad libitum*). All animal experimentation was carried out in the laboratory animal facilities (approval number: B-44-279). Experiments performed in this study were approved by the Ethics Committee for Animal Experimentation—Pays de la Loire Region, France (license N° CEEA 2012 171 for the biodistribution study, license N° CEEA 2013 2 for the RIT study). Animal caretakers that check the changes in behaviour, posture and appearance performed the monitoring of animals daily. The humane endpoints were a weight loss above 15% of initial weight or animals in a state of prostration. Animals that reach one of these endpoints were euthanized by cervical dislocation by caretakers. Blood sample collection was performed by retro orbital puncture under anaesthesia by isoflurane inhalation. In order to minimize animals suffering during blood samples collection, an eye drop of tetracaine 1% was applied. A table summarising the number of mice used in the study is presented in S1 Table.

### Radiolabelling, preparation and quality control of ^213^Bi-BSA and ^125^I-BSA

BSA was modified with 2-(4-isothiocyanatobenzyl)-cyclohexyl-diethylenetriaminepenta-acetic acid (SCN-CHX-A”-DTPA, Macrocyclics) as previously described [15]. In brief, BSA and non-specific antibodies were incubated with 20 equivalents (mol/ mol) of CHX-A”-DTPA in carbonate buffer (0.05 M, pH 8.7) overnight at room temperature and then purified by HPLC on a Sephadex G200 gel-filtration column (Amersham Biosciences, Saclay, France). The mean chelate number per BSA molecule was 2, as assessed with 4 equivalents of citrate-acetate (0.02–0.15 M, pH 5.5) buffered ^111^In solution. For labelling with ^213^Bi, the BSA-CHX-A”-DTPA was incubated with ^213^Bi eluted from a ^225^Ac/^213^Bi generator (Institute for Transuranium Elements, Karlsruhe, Germany) for 10 min in 0.8 M ammonium acetate (pH 5.3). The resulting ^213^Bi-labelled BSA was separated from unbound ^213^Bi by size-exclusion chromatography using a PD-10 column (GE Healthcare) [16]. Radiochemical purity, checked by ITLC-SG using 10% TCA as solvent [17], was greater than 95%.

BSA was labelled with ^125^I (Perkin Elmer, Courtaboeuf France) using the iodogen method as described previously [18]. The ^125^I-labelled BSA (^125^I- BSA) was purified on a PD10 column (GE Healthcare). Radiolabelling efficiency, estimated by ITLC, was above 95%.

### Biodistribution study and dosimetry

For biodistribution with ^125^I-BSA, mice were injected via the caudal vein with 5 μg of ^125^I- BSA (39.5 MBq/mg). Three mice were sacrificed at 15, 45, 90 and 180 minutes after injection. Different organs were collected and weighed. The amount of radionuclide activity in tissues was measured in parallel with a standard of injected activity using a gamma scintillation counter.

Biodistribution of ^213^Bi-BSA was performed following the same protocol. Mice were injected with 5 μg of ^213^Bi-BSA (346 MBq/mg). The decay of bismuth was corrected and adjusted to the time of sacrifice. The results were expressed as the mean percentage of the injected dose per gram of tissue (%ID/g) ± standard deviation (SD).

The absorbed doses to organs were estimated from the biodistribution data obtained with ^213^Bi-BSA by calculating the area under the curve according to the trapeze method with Prism software (Graph Pad software, San Diego, CA) in order to determine the total number of disintegrations in each organ for the considered time period for an arbitrary injected dose of 1 MBq. The dose to the organs per injected MBq was calculated by multiplying the total number of disintegrations by the mean energy of alpha particles. Beta and gamma emissions were not taken into account. The absorbed dose to organs of ^213^Bi deduced from the ^125^I-BSA biodistribution data was calculated after correction of ^125^I-BSA biodistribution for the decay of ^213^Bi. The same method of integration applied to ^213^Bi-BSA was used for the dose calculation of ^125^I-BSA biodistribution corrected for ^213^Bi decay.

### *In vivo* toxicological investigations

A single intravenous injection (caudal vein) was given of PBS to the control group (n = 5) and increasing activities of ^213^Bi-BSA: 3.7 MBq (n = 5), 7.4 MBq (n = 7) and 11.1 MBq (n = 5)). Survival and body weight were monitored weekly for 55 weeks. Blood was collected by retro orbital puncture into EDTA tubes in order to measure haematological parameters, and into lithium-heparin tubes in order to measure biochemical parameters. Counts of total white blood cells (WBC), red blood cells (RBC) and platelets were made on the day of injection and at weekly intervals thereafter for 35 days for control mice (n = 5) and for mice injected with 3.7 MBq (n = 5), 7.4 MBq (n = 7) and 11.1 MBq (n = 5) of ^213^Bi-BSA. The samples were counted on an MS9/5 vet haematology analyser (Melet Schloesing Laboratories, Osny, France). Biochemical parameters were measured using the plasma obtained after centrifugation of lithium-heparin blood samples. Blood urea nitrogen (BUN) and creatinine levels as well as aspartate aminotransferase (AST) and alanine aminotransferase (ALT) plasmatic activities were determined on the day of injection and at monthly intervals thereafter for 55 weeks on a Hitachi 917 Roche Analyzer (Meylan, France) using dedicated reagents. BUN was measured using the urease method, creatinine by the creatininase method, and AST and ALT using the International Federation of Clinical Chemistry method without pyridoxal phosphate. All biochemical parameters were expressed as the percentage ± SD of the counts relative to the baseline value (day 0, representing the day of the injection of ^213^Bi-BSA). Biochemical parameters were measured in control mice injected with PBS (n = 5) and in mice injected with 3.7 MBq (n = 5), 7.4 MBq (n = 7) and 11.1 MBq (n = 5) of ^213^Bi-BSA.

Survival curves were calculated using the Kaplan-Meier method and compared using the log rank test. Body weight, AST, ALT, BUN and creatinine curves were compared using the Mann-Whitney test. All analyses were 2-sided. P values of 0.05 or less were considered significant.

### Histological examination of mouse organs

Mice from the long-term follow-up were used for histological examination: control group (n = 5); 3.7 MBq group (n = 3); 7.4 MBq group (n = 4) and 11.1 MBq group (n = 2). Dedicated animals injected with 11.1 MBq (n = 4) were sacrificed 26 weeks after injection and mice injected with 3.7 MBq (n = 3) and 7.4 MBq (n = 4) were sacrificed 35 weeks after injection. Age-matched NMRI-nu mice from the same supplier (Janvier Labs, France) were used as historical controls (n = 3 and n = 2, respectively). Liver, kidney, lung, heart and spleen were fixed in 4% neutral-buffered formalin and processed by routine methods. Sections were stained with Haematoxylin-Eosin-Saffron (HES), Periodic Acid Schiff (PAS) and Masson’s Trichrome (MT) and evaluated with a Nikon Eclipse 5DI light microscope by two certified veterinary pathologists. The observations were graded as follows: 0 (absent), 1 (moderate), 2 (marked) and 3 (severe). Because of the number of animals, semi-quantitative variables (histological grade occurrence) of the different groups were compared using Fisher’s exact test. P values ≤0.05 were considered significant. R software (R development Core Team, Vienna, Austria) was used for the analysis.

## Results

### Pharmacokinetics and dosimetry

**Fig 1:**
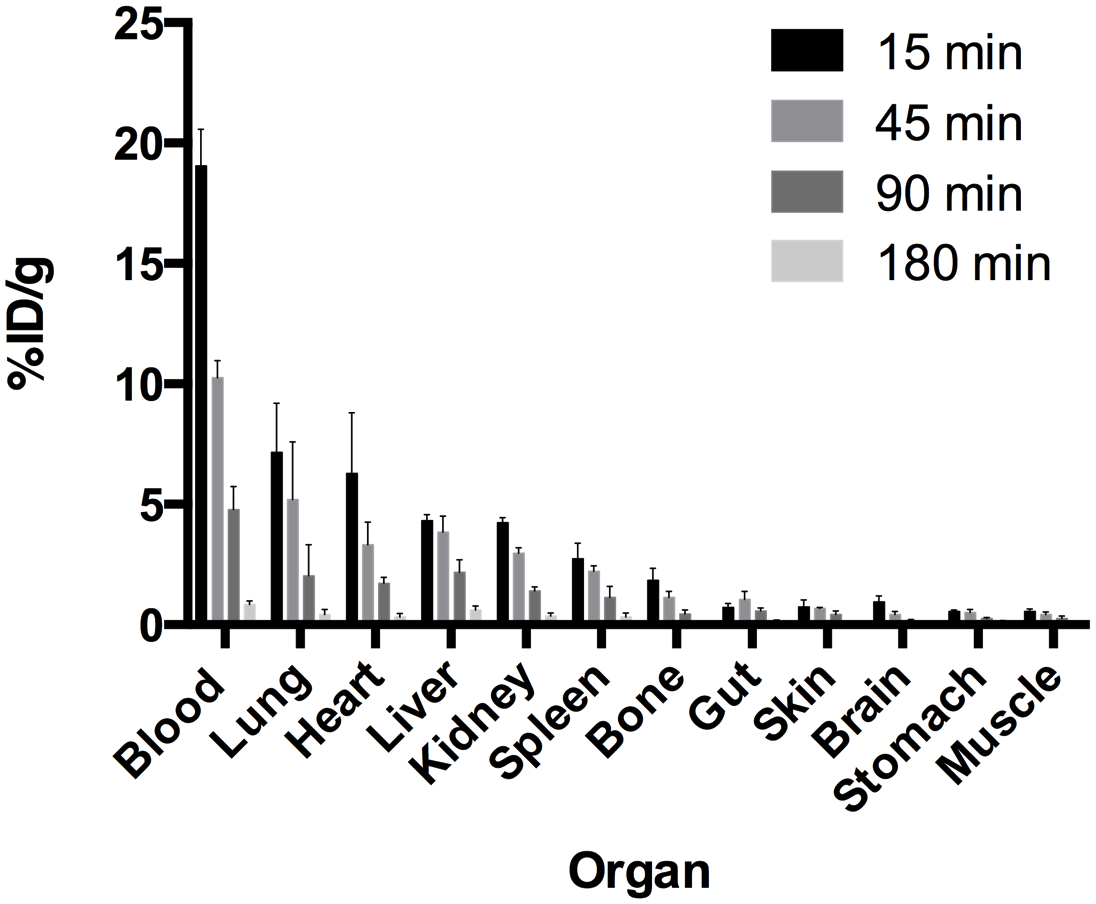
^213^Bi-BSA biodistribution in nude mice. Biodistribution of ^213^Bi-BSA was determined at 15, 45, 90 and 180 minutes after injection. Three mice were sacrificed at each time point. The results are expressed as the percentage of injected activity per gram ± SD.

The biodistribution of ^213^Bi-BSA was measured at 15, 45, 90, and 180 min after injection (Fig 1). The mean doses to organs deduced from the biodistribution data are given in Table 1. The highest mean absorbed doses were delivered to blood, lung, heart, liver and kidneys. Free bismuth is known to accumulate in the liver and kidneys and to be eliminated by faecal and urinary excretion [19]. The comparison of mean absorbed doses to organs deduced from ^213^Bi-BSA biodistribution with mean absorbed doses calculated by applying ^213^Bi decay to ^125^I-BSA biodistribution revealed the lowest dose accumulation in liver, kidney, spleen and heart and the highest doses in stomach and lung when ^125^I was used in place of ^213^Bi to radiolabel BSA (Table 1). In order to evaluate the contribution of ^213^Bi-BSA blood content and free ^213^Bi uptake to the mean absorbed dose to organs, organ to blood activity ratios obtained with ^213^Bi-BSA and ^125^I-BSA at different time points were compared. These ratios give insight into isotope accumulation in the different organs. A significant increase in these ratios was observed for liver, spleen and kidney for the biodistribution performed with ^213^Bi-BSA, in accordance with the known metabolism of proteins and the residualizing properties of bismuth-213. Conversely, the stomach to blood ratio increased for the biodistribution performed with ^125^I-BSA, consistent with the accumulation of free iodine in this organ due to the expression of iodine symporter (Table 2). No significant differences in organ to blood activity ratios were observed for other organs between ^213^Bi-BSA and ^125^I-BSA. The differences between ^213^BI-BSA and ^125^I-BSA mean doses to heart and lung may be related to their high blood content, which renders dose evaluation to organ tissues difficult to assess accurately.

**Table 1:** Mean dose to organs with ^213^Bi-BSA and comparison with mean dose to organs calculated applying ^213^Bi decay to ^125^I-BSA biodistribution.

	Gy/MBq	Gy/MBq	Gy/3.7 MBq	Gy/7.4 MBq	Gy/11.1 MBq
	^125^I-BSA	^213^Bi-BSA	^213^Bi-BSA	^213^Bi-BSA	^213^Bi-BSA
Blood	1.25	1.14	4.22	8.44	12.65
Liver	0.22	0.38	1.41	2.81	4.22
Kidney	0.26	0.30	1.11	2.22	3.33
Gut	0.09	0.09	0.33	0.67	1.00
Lung	0.56	0.49	1.81	3.63	5.44
Muscle	0.04	0.05	0.19	0.37	0.56
Spleen	0.15	0.22	0.81	1.63	2.44
Skin	0.10	0.07	0.26	0.52	0.78
Brain	0.04	0.05	0.19	0.37	0.56
Heart	0.29	0.38	1.41	2.81	4.22
Bone	0.13	0.11	0.41	0.81	1.22
Stomach	0.13	0.05	0.19	0.37	0.56

**Table 2:** Organ to blood activity ratio with ^125^I-BSA and ^213^Bi-BSA at different times.

	15min			45min			90min			180min		
	^213^Bi-BSA	^125^I-BSA	p	^213^Bi-BSA	^125^I-BSA	p	^213^Bi-BSA	^125^I-BSA	p	^213^Bi-BSA	^125^I-BSA	p
Liver	0.23 +/- 0.03	0.18 +/- 0.01	ns	0.37 +/- 0.05	0.16 +/- 0.01	****	0.45 +/- 0.04	0.19 +/- 0.03	****	0.72+/- 0.09	0.23 +/- 0.07	****
Kidney	0.22 +/- 0.03	0.21 +/- 0.03	ns	0.29 +/- 0,03	0.18 +/- 0.03	**	0.30 +/- 0.03	0.24 +/- 0.03	ns	0.23 +/-0.05	0.44 +/- 0.07	****
Spleen	0.14 +/- 0.04	0.15 +/- 0.04	ns	0.22 +/- 0.01	0.12 +/- 0.01	*	0.23 +/- 0.06	0.09 +/- 0.03	**	0.38 +/- 0.13	0.13 +/- 0.02	****
Stomach	0.03 +/- 0.003	0.06 +/- 0.01	ns	0.05 +/- 0.01	0.10 +/- 0.01	*	0.06+/-0.003	0.16 +/- 0.02	**	0.13 +/- 0.06	0.33 +/- 0.04	****
Heart	0.33+/- 0.11	0.21 +/- 0.04	ns	0.32 +/- 0.07	0.22 +/- 0.02	ns	0.36 +/- 0.04	0.27 +/- 0.02	ns	0.38 +/-0.14	0.33 +/- 0.13	ns
Lung	0.37 +/- 0.08	0.39+/- 0.09	ns	0.50 +/- 0.20	0.46+/- 0.15	ns	0.41 +/- 020	0.54 +/- 0.08	ns	0.51 +/-022	0.37 +/- 0.05	ns

The results are given as the mean +/- SD of organ to blood activity ratio at different times for each organ. Statistical significance between ^125^I-BSA and ^213^Bi-BSA organ to blood activity ratios was estimated with an unpaired t test:

*: p<0.01;

**: p<0.001;

***: p<0.0001;

****: p<0.00001).

### Toxicity: weight and survival

**Fig 2:**
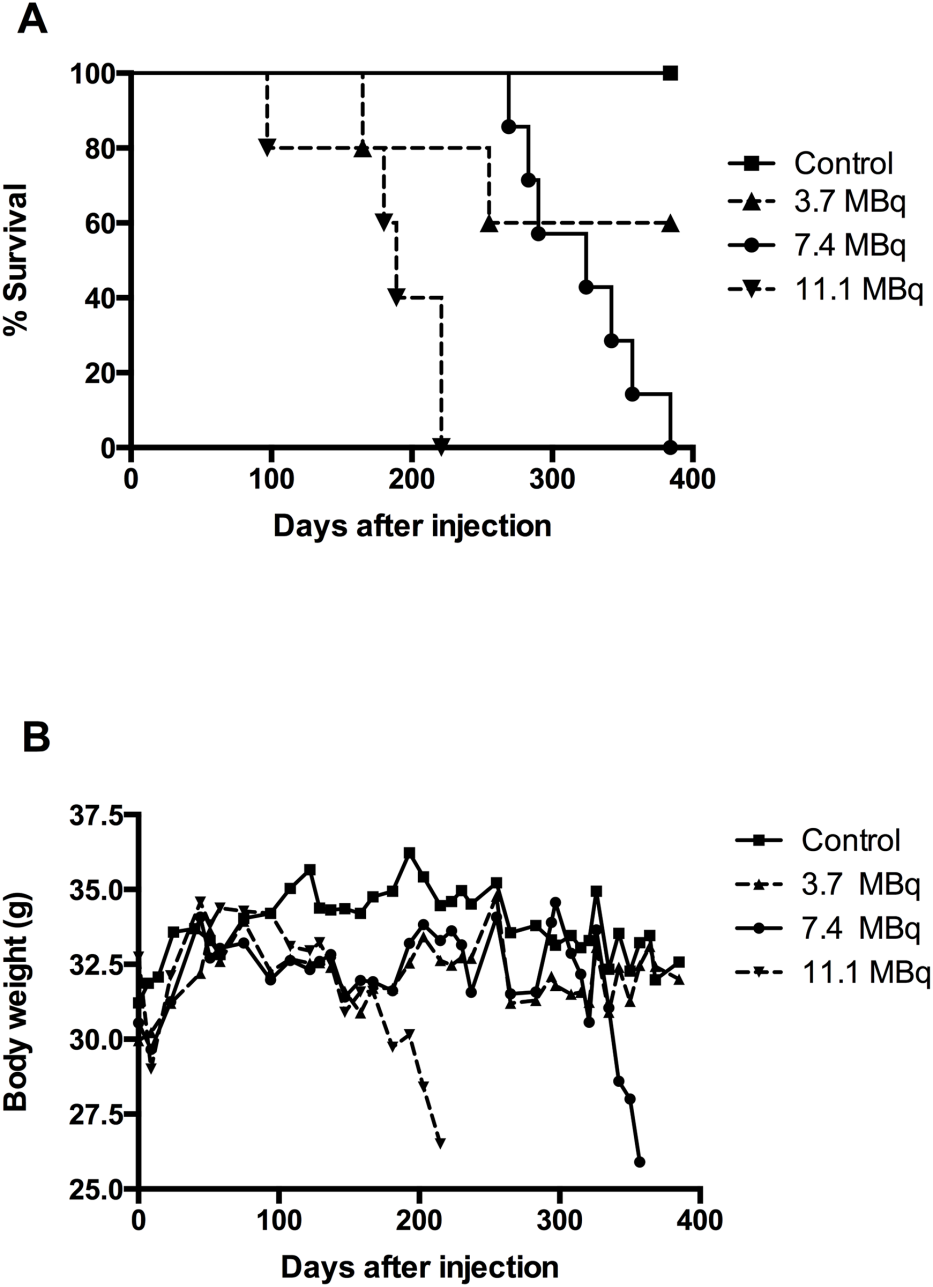
Total body weight and survival of mice injected with increasing activities of ^213^Bi-BSA. Three groups of mice were injected with ^213^Bi-BSA at increasing activities: 3.7 MBq (n = 5), 7.4 MBq (n = 7), and 11.1 MBq (n = 5) while a control group (n = 5) was injected with PBS. (A) In the 11.1 MBq injected group, the median survival period was 189 days. In the 7.4 MBq injected group, it was 324 days. In the 3.7 MBq injected group, one mouse died at day 165 and a second one at day 255. The median survival time was not reached in this group. All mice in the control group survived to the end of the assay. *: significant differences. (B) Total body weights were monitored over a 385-day period on the same animals. *: significant differences.

Three groups of nude mice were injected with PBS in the control group and with ^213^Bi-BSA at increasing activities: 3.7 MBq, 7.4 MBq, and 11.1 MBq. Body weight and survival were monitored every 9–10 days over a 385-day period (Fig 2A and 2B). In the 11.1 MBq injected group, all mice died between day 97 and day 221 with a median survival of 189 days. All mice injected with 7.4 MBq died between day 269 and day 357 with a median survival of 324 days. In the 3.7 MBq injected group (n = 5), one mouse died at day 165 and a second one at day 255 without prior weight loss or abnormal blood parameter and without obvious signs of suffering at daily monitoring. Autopsy was not feasible on these animals and we were not able to define the real causes of their death. All mice in the control group survived to the end of the monitoring period. Significant differences in survival were found between the control and the 7.4 MBq group (p = 0.0062), between the control and the 11.1 MBq group (p = 0.0014), between the 7.4 MBq and the 11.1 MBq group (p = 0.0005) and between the 3.7 MBq and the 11.1 MBq group (p = 0.034). No significant difference was found between the control and the 3.7 MBq group, or between the 3.7 MBq and the 7.4 MBq group (Fig 2A).

The body weight curves (Fig 2B) can be subdivided into several phases. During the first 10 days, all injected mice experienced a body weight loss less than or equal to 10% of their initial body weight. This first phase corresponded to acute and transient radiotoxicity since all mice recovered their initial weight between days 20 and 30 after injection, and continued to gain weight until day 75. No significant differences were observed between injected and control groups at this time. A subsequent drop in weight was observed for all injected animals compared to the control group up to day 150. Thereafter, differences between the injected groups began to appear. The weight curves of the 11.1 MBq group rapidly decreased concomitantly with the death of mice between day 97 and day 221. The weight of mice injected with 3.7 and 7.4 MBq stabilised during this period below the level of the control animals. Likewise for the 11.1 MBq group, the weight of mice of the 7.4 MBq group dropped between day 300 and day 350, during the period when death occurred. In the 3.7 MBq group, the weight of mice normalised and paralleled that of the control group.

### Haematological toxicity

**Fig 3:**
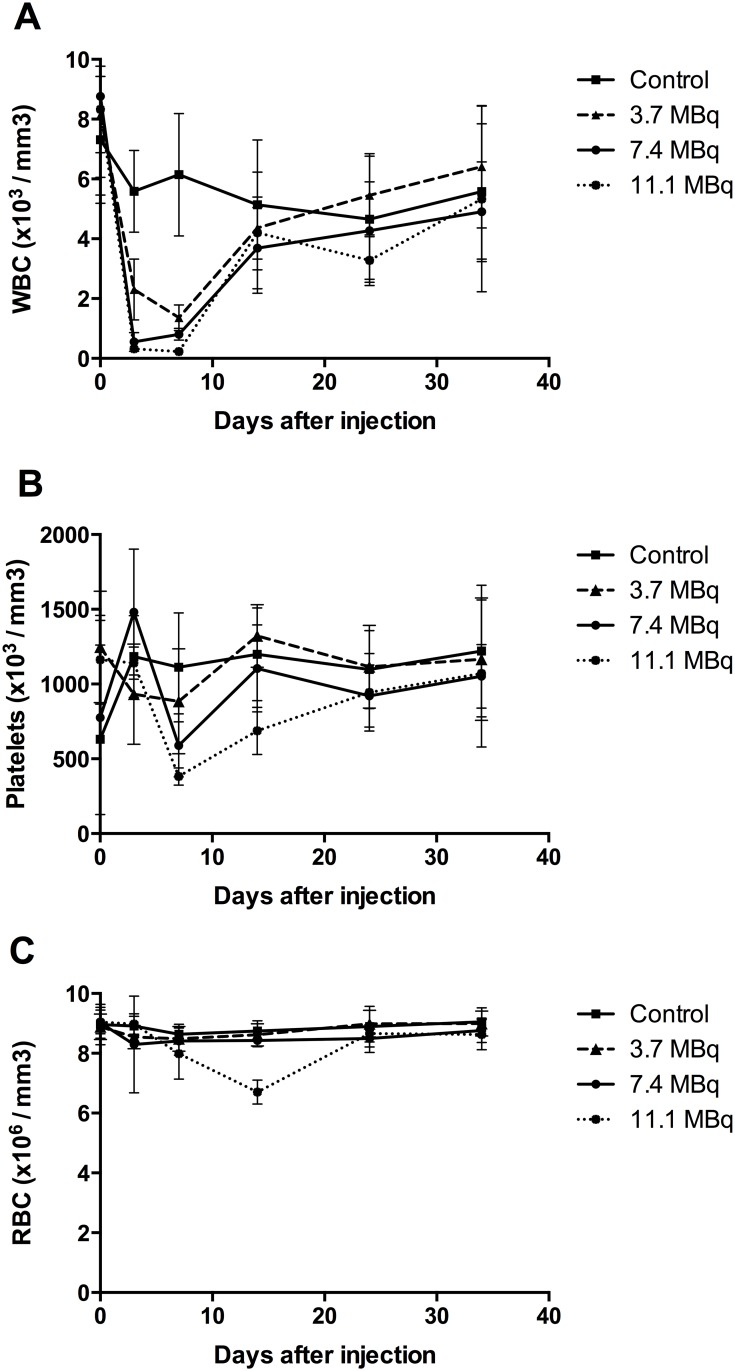
Haematological toxicity in mice injected with increasing activities of ^213^Bi-BSA. Blood counts of WBC (A), RBC (B), and platelets (C) were performed in mice injected with PBS, 3.7 MBq, 7.4 MBq and 11.1 MBq of ^213^Bi-BSA. The mean numbers (± SD) at each time point are shown.

Haematotoxicity was assessed by counting white blood cells (WBC), red blood cells (RBC) and platelets in control mice and mice injected with 0, 3.7, 7.4, and 11.1 MBq of ^213^Bi- BSA (Fig 3). Independently of the injected activity, haematological toxicity appeared rapidly after injection and was transient.

As early as three days after injection, a drop in WBC was observed, with a minimum at day 7. WBC decreased to 18.4%, 16.2% and 4.7% of the initial value (determined at day 0) for injected activities of 3.7, 7.4 and 11.1 MBq, respectively. A return to the initial WBC counts was observed at day 14 after injection for all injected activities (Fig 3A).

The minimum of the platelet count was reached at day 7 with 65.2%, 41.9% and 19.3% of the initial value for injected activities of 3.7, 7.4 and 11.1 MBq, respectively. A return to the initial number of platelets was observed at day 14 for the 3.7 and 7.4 MBq groups and at day 24 for the 11.1 MBq group (Fig 3B).

RBC counts remained unchanged for the 3.7 and 7.4 MBq groups but decreased to 48.7% of the initial value for the 11.1 MBq injected group at day 14, returning to the initial value at day 24 (Fig 3C).

Haematological data thus indicate that even the 11.1 MBq-injected activity was not myeloablative since mice injected with 11.1 MBq of ^213^Bi- BSA died between day 97 and day 221 with numbers of WBC, platelets and RBC comparable to those before injection and of the control group, indicating that toxicity to the bone marrow was not the dose-limiting toxicity [20].

### Analysis of hepatic and renal biochemical parameters

**Fig 4:**
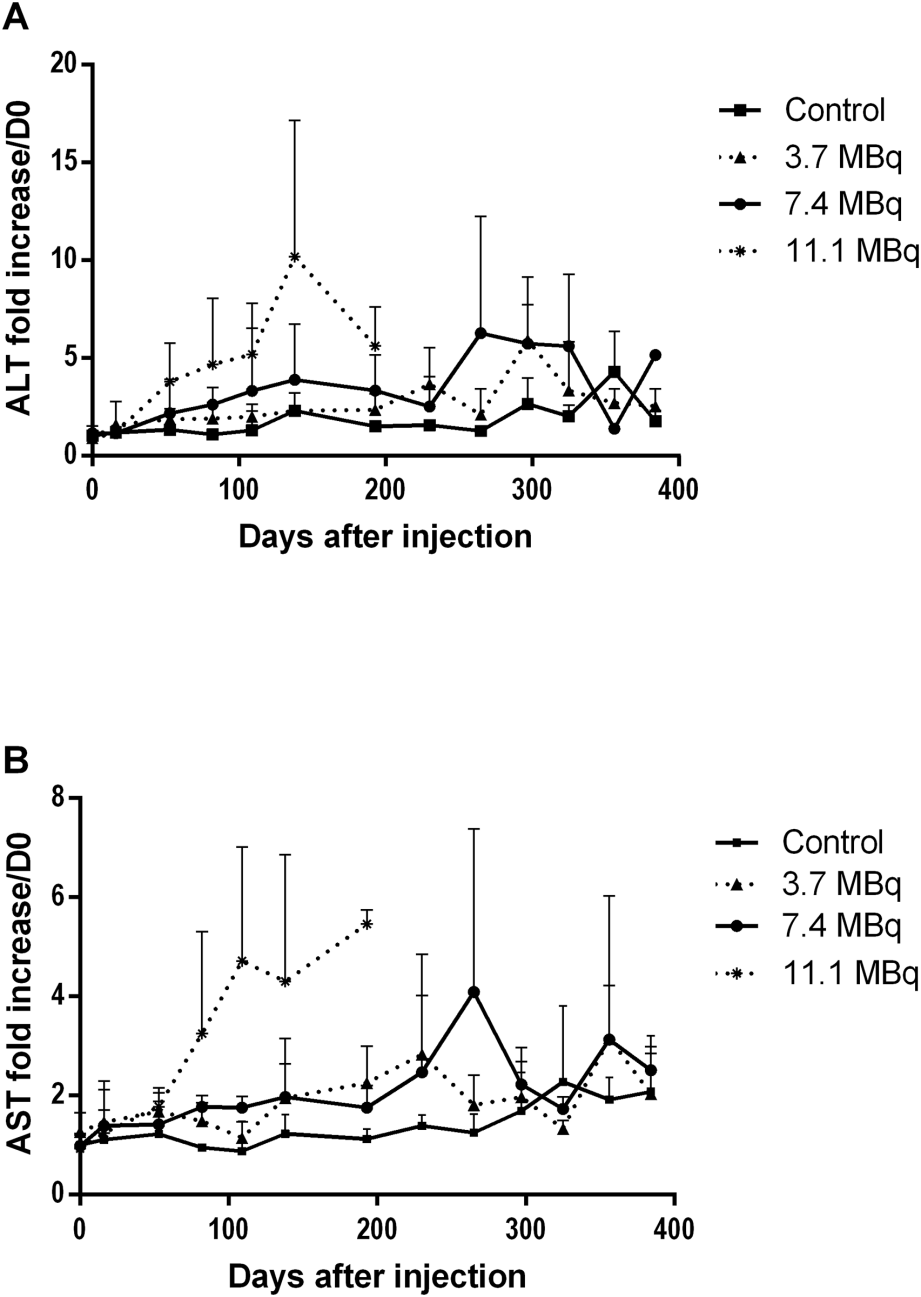
Monitoring of plasmatic ALT and AST for liver toxicity assessment. (A) ALT and (B) AST activity were assessed monthly in plasma of mice injected with PBS (control), 3.7 MBq, 7.4 MBq and 11.1 MBq of ^213^Bi-BSA. The mean level of ALT and AST (± SD) was expressed relative to the ALT and AST plasma activities at day 0 taken as a reference.

Plasmatic ALT and AST were measured to evaluate hepatic function since they reflect hepatic cytolysis. In the group of mice injected with 11.1 MBq, which died before day 220, an increase in AST (x3) and ALT (x5) was observed starting at day 100 and continuing to increase until death (x5 for AST and x10 for ALT). This indicated hepatocyte damage (necrosis or cytolysis), as ALT activity was higher than AST (Fig 4). For the two other groups, an increase in AST and ALT was also observed from day 250, which was more pronounced and sustained in the 7.4 MBq group than in the 3.7 MBq group.

**Fig 5:**
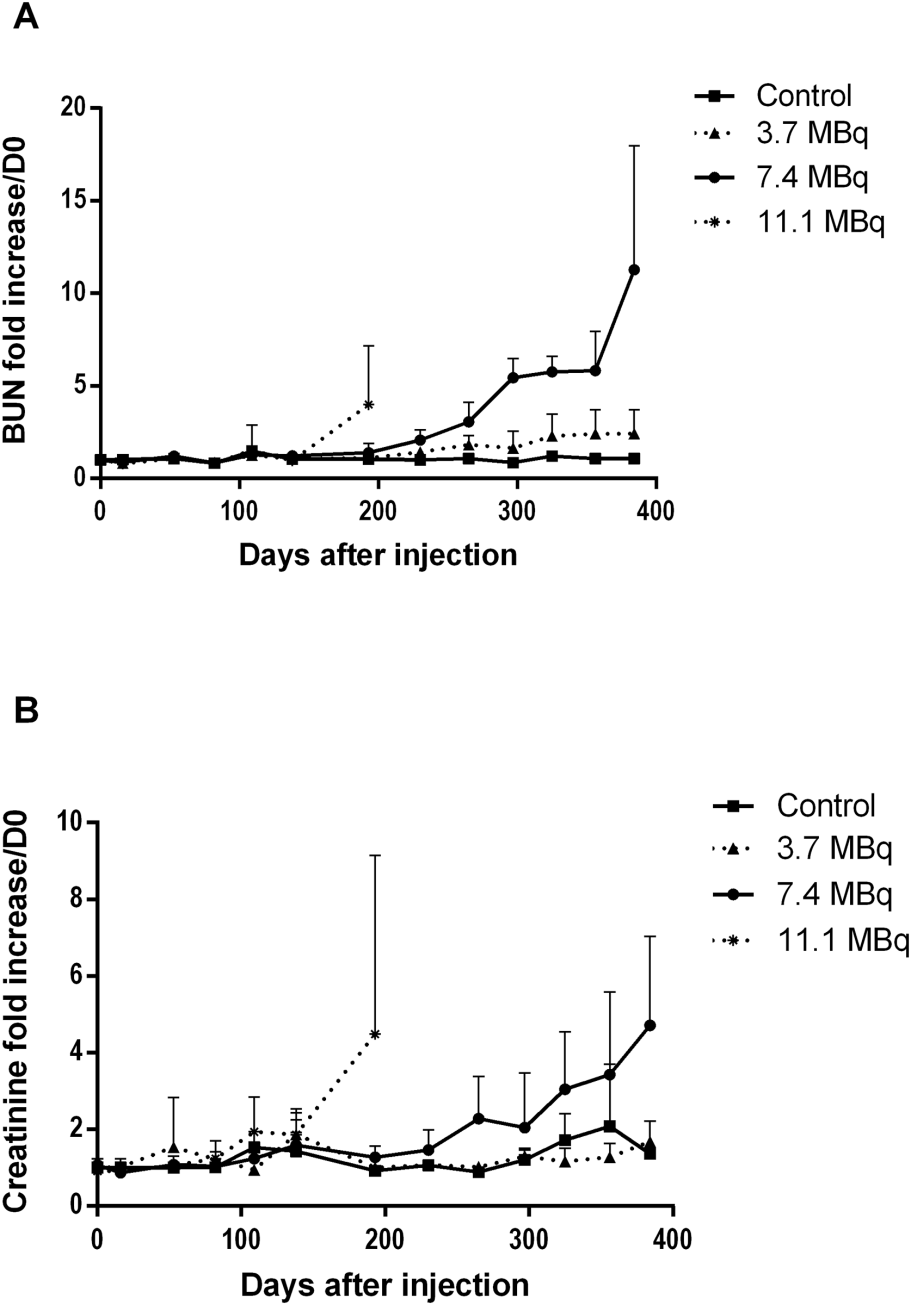
Plasmatic creatinine and blood urea nitrogen (BUN) monitoring for kidney toxicity assessment. (A) Creatinine and (B) BUN were assessed monthly in plasma of mice injected with PBS (control), 3.7 MBq, 7.4 MBq and 11.1 MBq of ^213^Bi-BSA. The mean level of creatinine and BUN (± SD) was expressed relative to the creatinine and BUN plasma levels at day 0 taken as a reference.

To evaluate kidney damage, blood urea nitrogen (BUN) and creatinine were measured (Fig 5). Uraemia is an indicator of kidney impairment but is also influenced by endogenous and exogenous catabolism. Serum creatinine represents a good indicator of the severity of renal impairment, particularly of GFR (glomerular filtration rate), as its plasma level increases when the functional nephron mass is reduced by 50%. Acute kidney injury (AKI) corresponds to a rapid loss of kidney function within the week after treatment. None of the injected mice, whatever the injected activity, showed an early elevation of azotaemia in the days after injection of ^213^Bi-BSA that would evidence AKI. The increase in azotaemia observed long-term after injection of mice with 11.1 and 7.4 MBq of ^213^Bi-BSA, (after 100 days and 200 days, respectively) is consistent with a chronic kidney injury (CKI). The severity of chronic kidney injury (CKI) is defined by the value of the glomerular filtration rate (GFR). In CKI, an increase in azotaemia is observed (creatininaemia and elevated BUN in plasma). Animals presenting a long-term elevated BUN and creatininaemia also present histological evidence of renal injury, most likely due to renal damage causing CKI. In the group of mice injected with 11.1 MBq, a parallel increase in uraemia and creatininaemia was observed before death, but not in all mice, contrary to the mice of the 7.4 MBq group, which all experienced a gradual and parallel increase in creatininaemia (x4) and uraemia (x5) (Fig 5) after day 250 consistent with chronic kidney injury (CKI). In the 3.7 MBq group, uraemia and creatininaemia remained low throughout the monitoring period, indicating that this injected activity was safe for the kidneys. For a more detailed analysis, a table summarising the time of animal death and the increases in blood parameters of liver and kidney is presented in S2 Table.

### Histological examination of the liver

**Fig 6:**
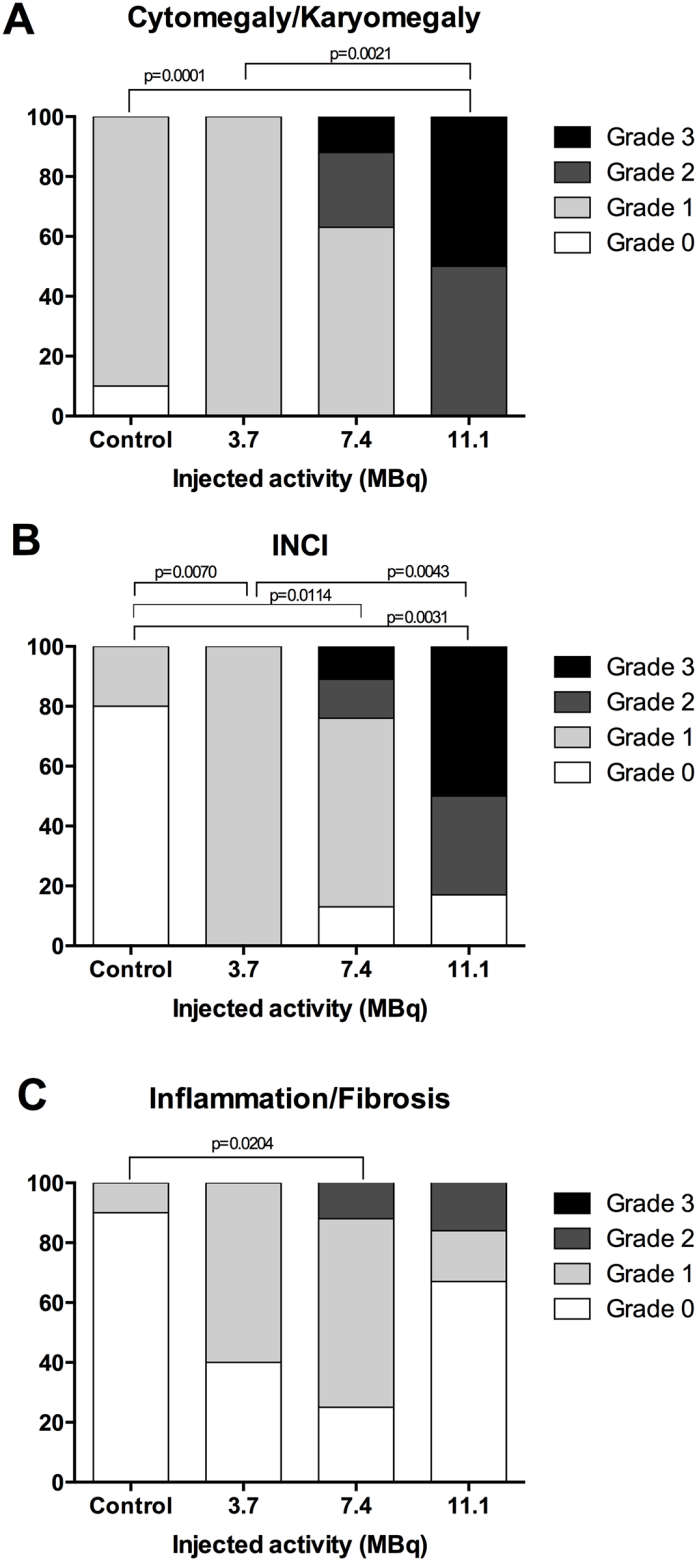
Grade of activity-related hepatic histological features. Activity-related increases in (A) cytomegaly/karyomegaly, (B) intranuclear cytoplasmic inclusion, and (C) inflammation/fibrosis of control mice (n = 10), mice injected with 3.7 MBq (n = 5), 7.4MBq (n = 8) and 11.1 MBq (n = 6). Histological features were graded on histological slides as follows: 0 (absent), 1 (moderate), 2 (marked) and 3 (severe). Histological grade occurrences in the different groups were compared using Fisher’s exact test. P values ≤0.05 were considered significant.

**Fig 7:**
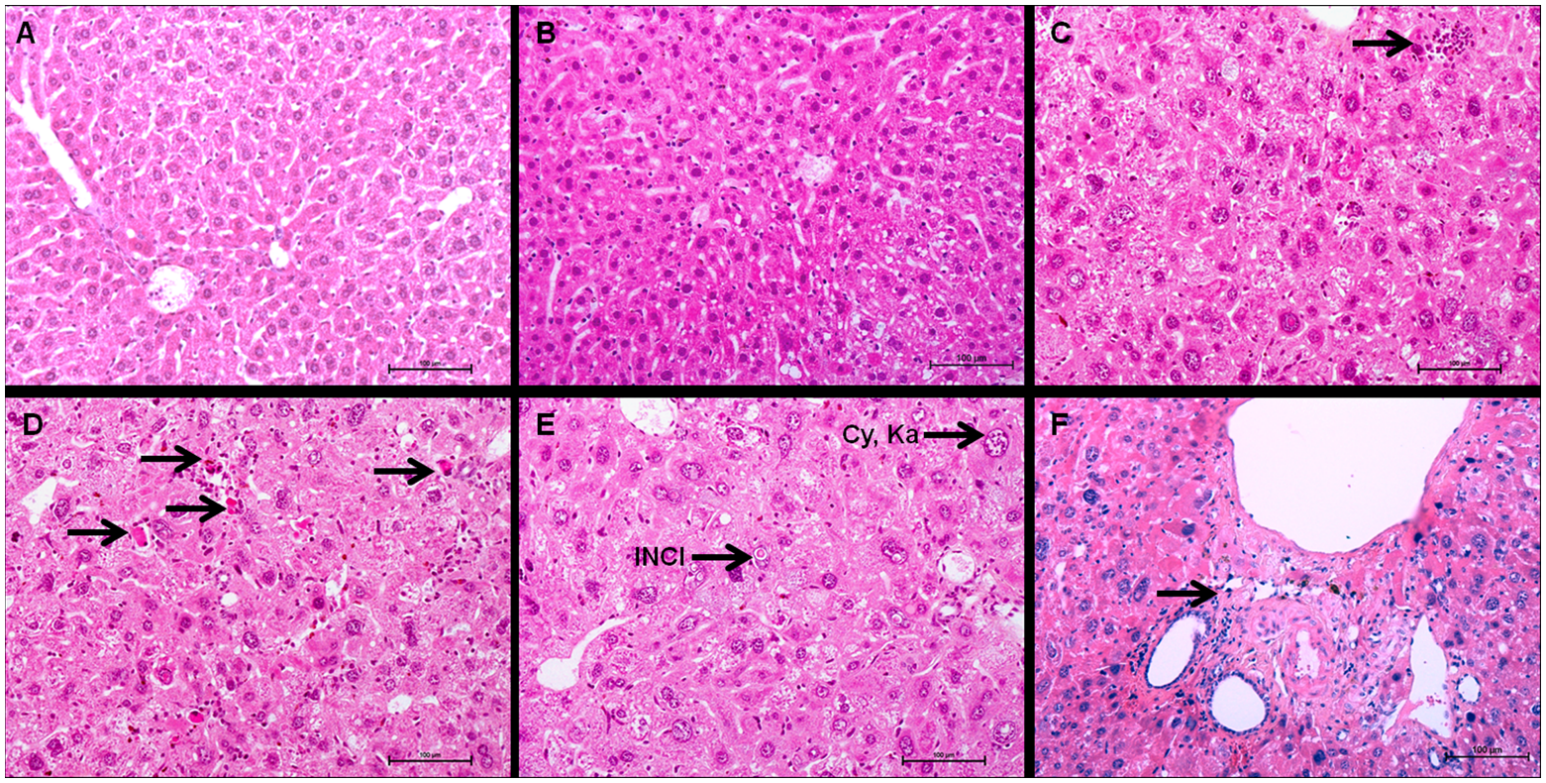
Liver histology of control and ^213^Bi-BSA injected mice. (A) Control liver, 26 weeks post-injection, (B) livers of mice 26 weeks after injection of 3.7 MBq ^213^Bi-BSA, and (C, D, E, F) livers of mice 26 weeks after injection of 11.1 MBq ^213^Bi-BSA. Livers of mice injected with 3.7 MBq ^213^Bi-BSA do not show any significant histological injury. Livers of mice injected with 11.1 MBq ^213^Bi-BSA show extramedullary haematopoiesis (C, arrow), isolated necrosis (D, arrow), karyomegaly (Ka), cytomegaly (Cy) and intranuclear acidophilic cytoplasmic invagination (INCI) (E). Inflammation and fibrosis are rarely observed (F, arrow). Haematoxylin-Eosin-Saffron (bar = 100 μm).

The liver of injected animals showed several microscopic changes. The severity of these changes increased with the injected activity. The more salient changes were cellular atypias (cytomegaly, karyomegaly, intra-nuclear cytoplasmic invagination (INCI), inflammation and fibrosis (Fig 6A, 6B and 6C). In rodents, extramedullary haematopoiesis (EMH) is commonly encountered in the liver and spleen in physiological conditions. In the liver, it is characterised by groups of dozens of haematopoietic precursors located in the space of Disse [21]. Extramedullary haematopoiesis is also encountered secondarily to radiation-induced myelotoxicity and failure of central haematopoiesis [21]. In our study, EMH did not significantly increase in mice injected with ^213^Bi-BSA compared to control groups (Fig 7C), and thus does not appear to be a major consequence of alpha irradiation in accordance with the limited myelotoxicity determined by blood cell enumeration. Activity-dependent increases of centrolobular fibrosis and periportal inflammatory cell infiltration indicated interstitial injury (Fig 7F). There was a statistically significant difference between the control and mice injected with 7.4 MBq (p = 0.0204) (Fig 6C). Isolated cellular necrosis was another indicator of hepatocyte injury. This was characterised by a small basophilic nucleus within a shrunken, acidophilic cytoplasm, often surrounded by a rim of degenerated neutrophils (Fig 7D). Although isolated necrosis seemed to be activity-dependent, no statistically significant difference could be found compared to the control group. The incidence and severity of cellular atypias (cytomegaly, karyomegaly and INCI) increased with injected activities. Cytomegaly is defined by the enlargement of the hepatocyte cytoplasm, due to an increase in the cytosolic protein content or number of organelles (e.g. smooth endoplasmic reticulum, peroxisomes, or mitochondria). It is generally considered an adaptive response to chemical stress. Karyomegaly is characterised by an increased size of hepatocyte nuclei and correlates with hepatocyte polyploidy, which occurs when there is duplication of nuclear material in the absence of cytokinesis (Fig 7E) [21]. The occurrence of these atypias was activity-dependent (Fig 6A). Intra-nuclear cytoplasmic invagination (INCI) corresponds to the protrusion of cytoplasm into an invagination of the hepatocyte nuclear membrane (Fig 7E). INCI is are common in the liver of aged mice but, in this study, there was a statistically significant difference between control and mice injected with 3.7 MBq (p = 0.0070), 7.4MBq (p = 0.0114) and 11.1MBq (P = 0.0031) (Fig 6B). These atypias were thus a consequence of a radiation-induced process. Finally, although histological changes can be observed in mice injected with 3.7 MBq of ^213^Bi-BSA compared to control animals (Fig 6B and 6C), the liver of mice from this group did not show significant histological changes that would indicate a major liver toxicity at this injected activity, in agreement with the maintenance of a basal level of ALT and AST up to the end of the study (Fig 4A and 4B) (S2 Table).

### Histological examination of the kidney

**Fig 8:**
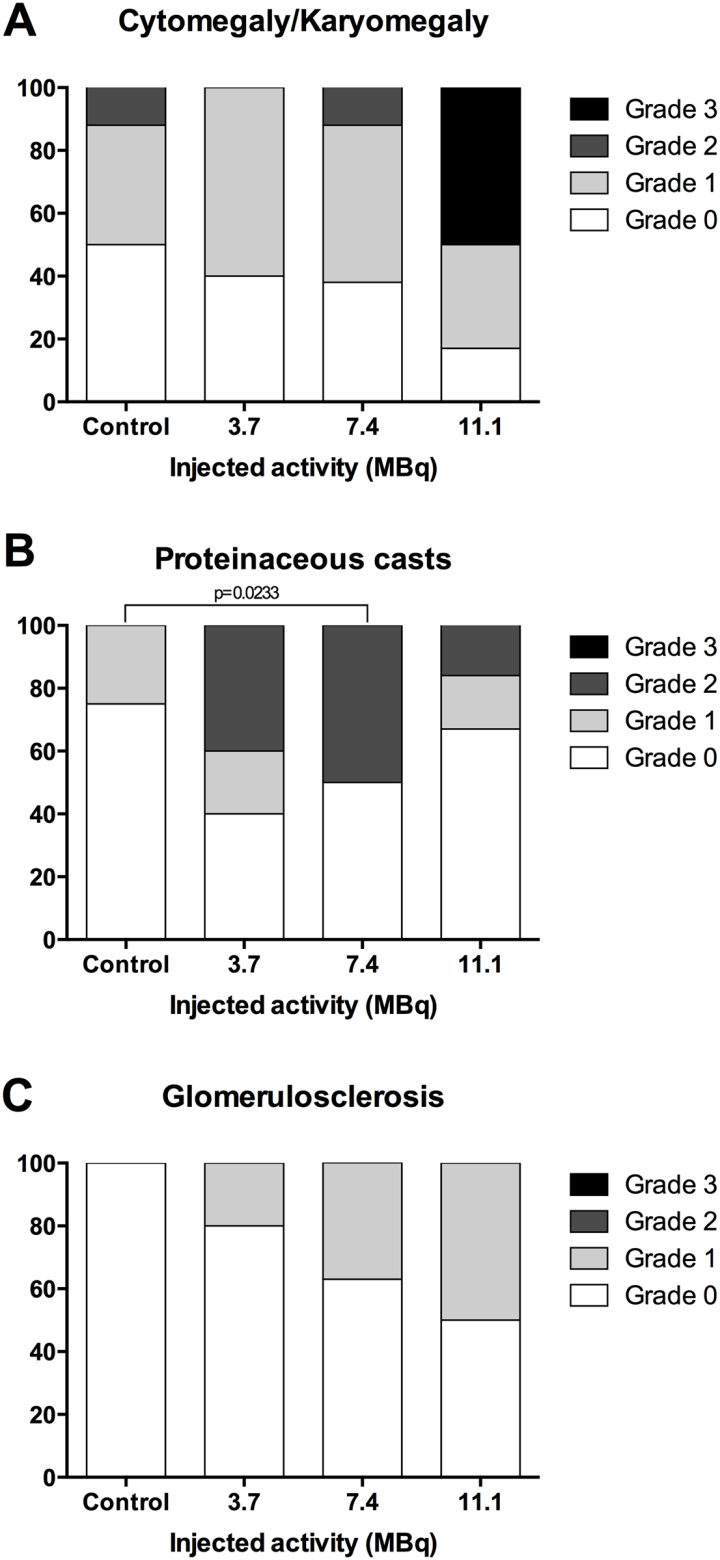
Grade of activity-related renal histological features. Activity-related increases in (A) cytomegaly/karyomegaly, (B) proteinaceous casts and (C) glomerulosclerosis, of control mice (n = 8), mice injected with 3.7 MBq (n = 5), 7.4 MBq (n = 8) and 11.1 MBq (n = 6). Histological features were graded as follows: 0 (absent), 1 (moderate), 2 (marked) and 3 (severe). Histological grade occurrences in the different groups were compared using Fisher’s exact test. P values ≤0.05 were considered significant.

**Fig 9:**
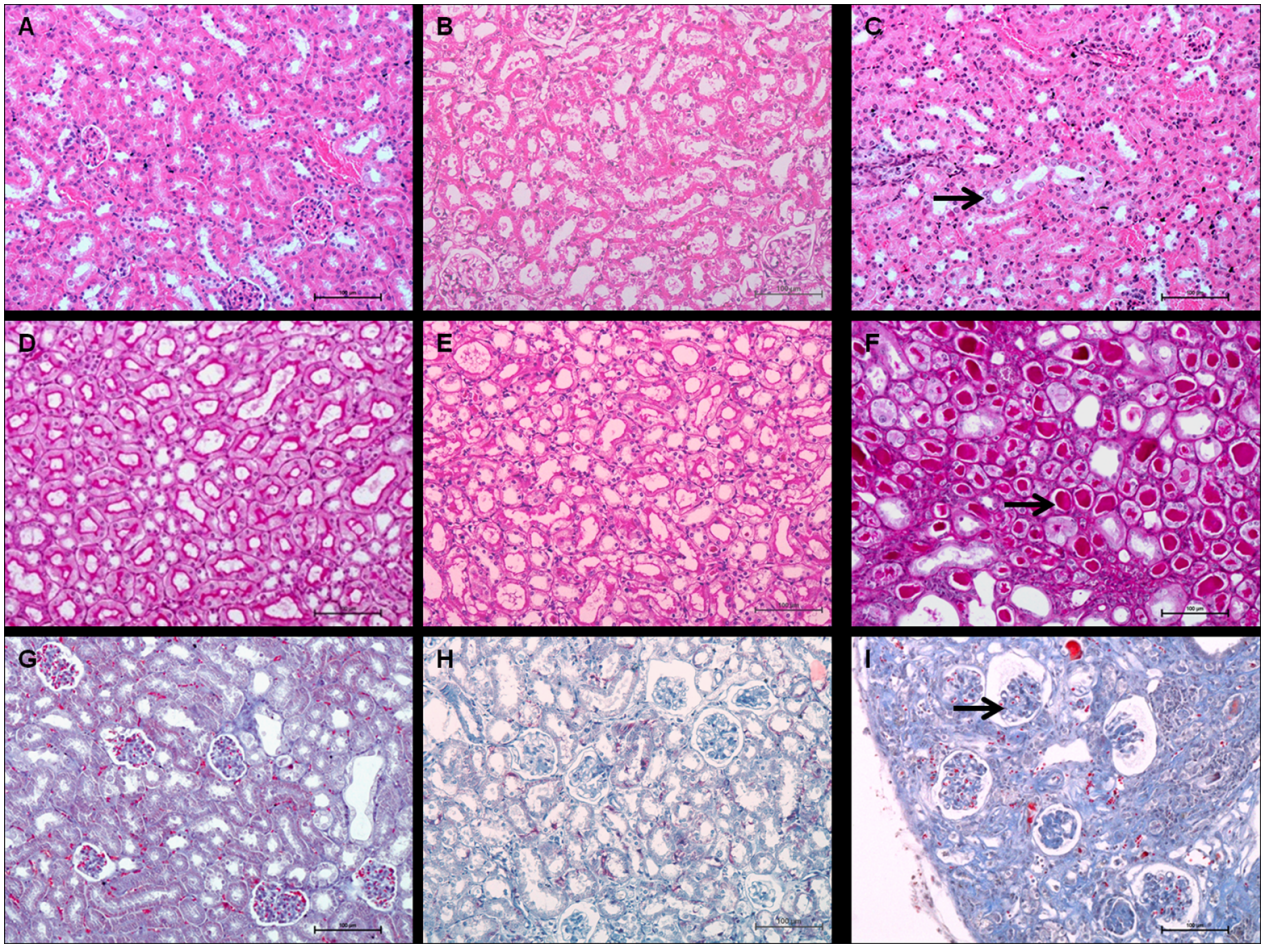
Kidney histology of control and ^213^Bi-BSA injected mice. Comparison between kidneys of (A, D, G) control animals, (B, E, H) kidneys of mice 35 weeks after injection of 3.7 MBq of ^213^Bi-BSA, (C and I) 26 weeks after injection of 11.1 MBq of ^213^Bi-BSA and (F) 35 weeks after injection of 7.4 MBq of ^213^Bi-BSA. Control kidneys, Haematoxylin-Eosin-Saffron (A), Periodic Acid Schiff (D), and Masson’s Trichrome (G). Kidneys of mice injected with 3.7 MBq ^213^Bi-BSA do not show any significant histological injury (B (HES), E (PAS) and H (MT)). Kidneys of mice injected with 11.1 or 7.4 MBq ^213^Bi-BSA show basophilic tubules, karyomegaly and cytomegaly (C, arrow) (HES), proteinaceous casts (F, arrow), (PAS), and glomerulosclerosis (I, arrow) (MT) (bar = 100 μm).

Kidney examination revealed several histological anomalies. The severity of these cellular atypias (cytomegaly, karyomegaly), proteinaceous casts and glomerulosclerosis increased with the injected activity (Fig 8A, 8B and 8C). Basophilic tubules, cytomegaly, and karyomegaly suggested regenerative changes (Fig 9C).

Tubular injuries were also identified through wrinkled and thickened basement membranes, demonstrated by PAS stain. Dilation of Bowman spaces and some cortical tubules occurred secondarily to tubular loss or tubular obstruction due to proteinaceous casts. All these findings characterise tubular injury and their frequency and severity increased in an activity-dependent manner, though no significant differences between control and injected mice could be shown. Glomerular injury was characterised by capsular and mesangial sclerosis and gave rise to proteinaceous casts, visible by PAS staining (Fig 9F). Glomerular injuries were also identified through wrinkled and thickened basement membranes demonstrated by PAS staining. Some moderate interstitial lesions were recorded, such as moderate inflammatory cell infiltration and fibrosis (Masson’s Trichrome) (Fig 9I). Similarly to tubular injury, glomerular injury seemed to be activity-dependent although no statistically significant difference between groups could be shown. This is probably due to the limited sample size and the setting of histological analysis, which was performed on mice dedicated to histopathological analysis at specific follow-up times irrespective of biochemical evidence of kidney failure, which occurred late in the follow-up period. In the group of mice injected with 3.7 MBq of ^213^Bi-BSA, the histological changes were limited compared to control mice (Fig 8B, 8E and 8H). These histological changes did not seem to impair kidney function, at least during the long-term follow-up, in agreement with the limited increase in BUN detected in the mice of this group at the end of the assay (Fig 5A and 5B) (S2 Table).

### Histological examination of lung, heart and spleen

Lung, heart and spleen showed no histological alteration. Classic X-ray radiation-induced injuries, such as pulmonary radiation fibrosis, vascular changes in heart, lymphoid depletion in the white pulp of the spleen, or diffuse fibrosis of the red pulp, were not observed.

### Relationship between histological and biochemical analysis

Even though statistical correlations were not always found between the injected activity and histological observations, the biochemical data of hepatocyte cytolysis (activity-dependent increase in AST and ALT) were consistent with hepatocyte and interstitial injury at the highest injected activity. Similarly, despite not reaching statistical significance in some cases, histological modifications observed in the kidney (dilation of Bowman spaces, glomerulosclerosis, intratubular proteinaceous casts) were consistent with the increase in blood creatinine and BUN levels, clearly suggesting impaired renal function at 7.4 MBq injected activity due to tubular and glomerular injury.

## Discussion

The tolerance dose of normal tissues to whole organ volume X-ray irradiation is well established and ranks organs from the most to the least radiosensitive as bone marrow, lung, kidney, heart and liver [22]. This study of body irradiation by systemically administered ^213^Bi-BSA gives a different scheme of long-term toxicity, with kidney and liver being more radiosensitive than lung and heart on the basis of biochemical parameters and/or histological analysis. The toxicity to bone marrow induced by ^213^Bi-BSA is transient and not limiting at the injected activities used in this study. This pattern of long-term toxicity is not linked to the mean absorbed dose to organs since the lung received the highest dose (0.49 Gy/MBq) followed by the liver and heart (0.38 Gy/MBq) and kidney (0.30 Gy/MBq). Thus, the mean absorbed dose to the organ was insufficient to take into account the specificity of high-LET alpha particle irradiation. While the energy deposit is homogenous for X or gamma rays, the actual energy deposition to an organ for a given mean absorbed dose of high-LET radiation is influenced by the location of dose deposition at the cellular scale. It is beyond the scope of this article to provide a microdosimetric modelling of an alpha irradiation of mouse organs from the blood volume, but the unexpected radioresistance of lung to alpha particles compared to its high radiosensitivity to external beam irradiation underlines the necessity to investigate dose deposition at the cellular level in order to link organ damage to injected activity.

As far as lung is concerned, the tridimensional distribution of cells and their effective probability of being crossed by alpha tracks may explain their lower radiosensitivity to alpha particles for at least three simple reasons. Firstly, because of its alveolar structure, the density of the lung is one quarter that of the liver so that the dose to organ takes into account the energy of the alpha particles lost in the air volume, contrary to other organs where virtually all the energy is deposited in cells. Secondly, the distance between cells and blood capillaries and the density of the vascular network per volume of tissue is lower in lung compared to liver. Thirdly, the cell shape influences the quantity of energy deposited by an alpha particle. As an example, the energy deposited by an alpha track crossing a type I pneumocyte is limited because of their flat shape with a cytoplasm covering the alveolar surface as a sheet of around 0.4 μm in thickness.

Similarly, the histological structure of the bone marrow may explain the limited toxicity of alpha particles. Although myelotoxicity is a concern in radioimmunotherapy, the kinetics of WBC, RBC and platelet reconstitutions after ^213^Bi-BSA irradiation are very fast and correspond to the time required for each haematopoietic population to differentiate and be exported from the bone marrow to the periphery. The minimum number of platelets and leucocytes appeared to be independent of the injected activity but, at 11.1 MBq, the recovery of a normal platelet count was delayed (24 days vs. 14 days for 3.7 and 7.4 MBq injected activities). This could reflect a partial destruction of the bone marrow stroma, which would impair central haematopoiesis and correlate with the appearance of extramedullary haematopoiesis in the liver at injected activities higher than 3.7 MBq.

Watchman et al. clearly showed that a large number of bone marrow haematopoietic stem cells are located further than 70 μm from the nearest blood vessel [20]. Even if some ^213^Bi-BSA leaves the blood flow and moves by convection into the interstitial space, it is likely that a sufficient number of stem cells located far from a blood vessel are protected from alpha irradiation and able to repopulate the bone marrow. In fact, following total body irradiation, as little as 5% of the pool of stem cells is sufficient to reconstitute haematopoiesis successfully [23]. This observation of a limited alpha RIT myelotoxicity is consistent with previous long-term toxicity assays in rodents that point out a limiting toxicity to kidneys.

In our long-term toxicity assay, we also observed a significant increase in BUN and creatinine at 7.4 and 11.1 MBq injected activities, corresponding to a mean absorbed dose to kidney of 2.22 and 3.33 Gy, respectively. This increase in BUN and creatinine is concomitant with the death of animals. We thus confirm in this assay that toxicity to kidney is limiting for alpha RIT applications. However, in our assay, this toxicity to kidney is observed at a low mean dose compared to previous studies. Using ^213^Bi-PAI2 as a radiolabelled vector, Song et al. observed kidney damage at a mean dose to kidney of 11.5 to 14.4 Gy leading to death at 23 and 24 weeks, respectively, with no sign of toxicity to other organs [5]. In line with this finding, Behr et al. using ^213^Bi-CO17-1A Fab’ defined the MTD at 25.9 MBq and concluded that the corresponding dose of 54 Gy to kidney seemed to be well tolerated although 35% of the mice died after 6 months [6]. Using ^213^Bi radiolabelled peptides, a dose of 6 and 11 Gy to kidney with ^213^Bi-DOTA-PESIN and ^213^Bi-AMBA, respectively, led to tubular degeneration and karyomegaly 20 weeks after treatment, with recovery 10 weeks later due to the regeneration potential of the renal tubular epithelium [7].

A major difference between these assays and ours is the use of small vectors with a size under the kidney filtration threshold. With this kind of vector, blood clearance is fast and most of the radiolabelled vector is eliminated by the renal route during the time of ^213^Bi decay, leading to high doses to kidney.

Whereas the mean dose to blood with small vectors remains under 0.3 Gy/MBq, the mean dose to blood calculated with ^213^Bi-BSA is 1.14 Gy/MBq. Because of the fast filtration of the small vectors and rapid decrease of the blood dose, the irradiation of kidney glomeruli from the blood should be shortened compared to tubular irradiation. Conversely, larger vectors with a size above the kidney filtration threshold, like ^213^Bi-BSA, may tend toward a stronger irradiation of glomeruli compared to tubules due to their lasting presence in the blood. Reabsorption of radiolabelled vector by the proximal tubule may also be limited in the case of large vectors compared to small ones and thus the dose to tubules is probably lower for high molecular weight vectors. This difference in the local irradiation of the nephron substructure may partly explain the toxicity observed at a low mean dose to the kidney with ^213^Bi-BSA in our study compared to smaller ^213^Bi-radiolabelled vectors.

Another non-exclusive explanation of toxicity at the low dose to kidney in our assay may be related to the length of the follow-up. In fact, it has been shown that toxicity to kidney is delayed when the injected dose decreases. Song et al., studying the toxicity of ^213^Bi-PAI2 in mice, noted that in a 13-week follow-up period, the MTD deduced from weight loss was up to 1420 MBq/kg [5]. During this period, damage to kidney was mild but mice asymptomatic at 13 weeks become symptomatic at 20 weeks and reached the end-point at 30 weeks. A similar influence of the dose on the time of renal toxicity occurrence was observed in a long-term toxicity study by the team of Back et al. measuring glomerular filtration after injection of an ^211^At-labelled Fab’(2) in non-tumour-bearing mice. A 50% reduction in glomerular filtration was observed at 8–30 weeks for animals receiving a mean dose to kidney of 14 Gy, but 11 Gy to kidney was sufficient to induce the same glomerular filtration reduction in the period of 31–61 weeks. They also observed a progressive reduction in glomerular filtration a long time after injection (42–50 weeks) for doses to kidney above 3 Gy [2]. The age of mice at the time of kidney failure should thus be considered in the dose-toxicity response. In our assay, in the 7.4 MBq group, corresponding to a mean dose to kidney of 2.22 Gy, all mice died between weeks 38 and 51 with a huge increase in creatinine and BUN and histological changes revealing kidney damage. The physiological changes in kidney structure and function with aging resulting in a decreased glomerular filtration rate might also be considered when comparing the dose-toxicity relationship [24, 25]. A partial destruction of the functional glomeruli with a mean dose to kidney of 2–3 Gy would leave enough intact nephrons to ensure renal function in young animals but would hasten kidney senescence in aging mice, leading to precocious kidney failure.

A third characteristic of alpha RIT toxicity with high molecular weight radiolabelled vectors like BSA, IgG or Fab’(2) IgG fragment is the high mean dose to the blood (1.14 Gy/MBq to kidney with ^213^Bi-BSA). Consequently, the use of a large vector with a size over the kidney filtration threshold and with a liver elimination route leads to a sustained irradiation of healthy organs from the blood and to a particular pattern of toxicity, as illustrated by this study and by previous assays in rodents, dogs and humans using ^213^Bi-radiolabelled antibodies in the context of a conditioning regimen for haematopoietic cell transplantation or in the treatment of myeloid leukaemia.

Using a ^213^Bi anti-CD45 antibody in a mouse model, Nakamae et al. [11] detected a huge peak of AST and ALT as early as three hours after antibody injection at 2.57 Gy delivered to the liver, attesting an acute phase of cytolysis. AST and ALT then returned to baseline levels one week after RIT and during the eight weeks of their follow-up period. We did not observe this acute toxicity because, in our follow-up setting, the first measurement of AST and ALT took place two weeks after injection. At this stage, ALT and AST were at the control level regardless of the injected activity as in the Nakamae study. ALT and AST in the plasma remained at the control level until weeks 7 and 11, respectively, when a significant increase was observed in mice injected with 11.1 MBq of ^213^Bi-BSA corresponding to 4.22 Gy to the liver. At this dose, liver functions were irreversibly impaired. We did not observe central vascular injury with central congestion and venous fibrosis, referred to as veno-occlusive disease (VOD) and characteristic of X-ray late toxicity to the liver. However, sparse centrolobular fibrosis and periportal inflammation observed at the highest injected activity are consistent with a limited interstitial injury. Cellular atypias and progressive increases in plasma enzymes ALT and AST, at the highest ^213^Bi-BSA doses, clearly demonstrated radiation-induced toxicity to the liver.

Our results are consistent with those observed for alpha RIT in conditioning regimens for haematopoietic cell transplantation in dogs treated with ^213^Bi-anti-CD45 [26] or ^213^Bi-anti-TCRαβ chain antibody [27] and in humans for anti-leukemic treatment with ^213^Bi-anti-CD33 [28]. In dogs, a transient increase in ALT and AST consistent with liver toxicity was observed for injected activities of ^213^Bi-anti-CD45 between 133 and 170 MBq/g and one dog treated with 326 MBq/kg was euthanized because of liver failure [26]. In another assay, dogs treated with ^213^Bi-anti-CD45 exhibited more pronounced liver toxicity at injected activities comprised between 122 and 180 MBq/kg. Lastly, Bethge et al. using ^213^Bi-anti-TCRαβ for conditioning for allogeneic canine marrow transplantation reported a transient AST and ALT increase for injected activities comprised between 137 and 207 MBq/kg and a sustained level of these enzymes to the end of the study in one dog receiving 167 MBq/kg. Interestingly, like in mice, these increases in liver enzymes appeared between days 40 and 100. Histological examination of the liver of dogs receiving the highest doses of anti-CD45 or anti-TCRαβ revealed slight signs of sinusoidal fibrosis and minimal bile duct abnormalities and, similar to mice, were not consistent with radiation-induced VOD and liver fibrosis following high doses of X-rays. In humans receiving ^213^Bi-anti-CD33 for RIT for myeloid leukaemia, although injected activities were ten times lower than in dogs (ranging from 10.36 to 37 MBq/kg), the only adverse effect reported, except for the haematological toxicity, was a transient liver function abnormality in one third of patients between days 5 and 14 [28]. Another clinical trial combining Cytarabine and RIT with ^213^Bi anti-CD33 treatments was performed on thirty-one patients receiving 37 to 46.25 MBq/kg. Twenty-one patients developed a transient increase in bilirubin alkaline phosphatase and transaminase between days 3 and 30 and five in this group developed grade 3 or 4 liver abnormalities [29]. This is consistent with an early increase in AST and ALT in mouse soon after injection of ^213^Bi-radiolabelled antibody reported by Nakamae et al. at the dose of 2.57 Gy to the liver (1.85 MBq rat anti-mouse ^213^Bi anti-CD45 Mab). Given the scale differences between the different species and the specificity of the ^213^Bi-Mab used in these assays, it is interesting to compare the doses to liver between species.

In this long-term toxicity study in mice, liver toxicity was observed in the group of mice receiving 11.1 MBq (343 MBq/kg), corresponding to a 4.22 Gy dose to the liver, whereas only some mice injected with 7.4 MBq (228 MBq/kg), corresponding to 2.81 Gy to the liver, experienced a transient increase in transaminases. In assays of conditioning regimen for allogeneic marrow graft with either ^213^Bi-anti-TCRαβ or ^213^Bi-anti-CD45 in dogs, the dose to liver was not specifically calculated for the treated animals. However, in the assay of Bethge et al., a dose estimate of between 1.8 and 4.2 Gy to liver can be extrapolated from the dosimetric analysis of the dose to organ performed on one dog injected with ^213^Bi-anti-TCRαβ. In this assay, an increase in transaminase and alkaline phosphatase occurred between day 30 and day 100 but the plasma level of enzymes returned to the baseline for three dogs out of four. At autopsy, the dog with sustained high transaminase had histological liver changes in the form of pericellular and periportal fibrosis although no signs of frank cirrhosis were observed at the time of examination [27].

In human clinical trials, in an article dealing more specifically with the pharmacokinetics and dosimetry [30] of ^213^Bi-labelled anti-CD33 antibody used in a leukaemia RIT clinical trial, doses are calculated from images and are given in Sv rather than Gy because a multiplication factor of 5, corresponding to the RBE of alpha particles, is included in the dose calculation. The mean dose to the liver can be estimated as 5.21 ± 1.58 mSv/MBq, which corresponds to a mean dose of 1.05 mGy/MBq without RBE. A patient of 65.9 kg (mean patient weight in this study) would thus have received 2.56 Gy to the liver for an injected activity of 37 MBq/kg, which corresponds to the threshold of liver damage observed with the ^213^Bi-labelled anti-CD33 antibody. Thus, the doses to liver generating a risk of irreversible liver failure appear quite similar in mice (this study), dogs and humans. In dogs and humans, no obvious signs of kidney failure were observed during the follow-up.

## Conclusion

This study emphasises the difference in toxicity patterns observed between small vectors like peptides, with a size under the kidney filtration threshold, and larger macromolecules, with a size over this threshold, like antibody, Fab’(2) Ig fragment or BSA, used in this study as an example of a long-lasting plasmatic protein. The liver route of catabolism of these macromolecules together with the accumulation of free bismuth in this organ induces liver toxicity. Conversely, the fast elimination of smaller vectors by the renal route and the reabsorption of radiopharmaceutical in proximal tubules deposit high doses to kidney while blood and liver doses remain under the toxic limit.

In our long-term toxicity trial in mouse, we also observed kidney toxicity a long time after injection at a relatively low dose to kidney, contrary to what has been observed in dogs and humans treated with ^213^Bi-radiolabelled antibody. This discrepancy in toxicity between mice and humans or dogs may be related to the length of the follow-up period compared to the life span of each species. In mice, the natural decrease in glomerular filtration with aging during a year of follow-up, corresponding to a third of their life expectancy, would sufficiently impair kidney function for radiation-induced glomerular filtration to become limiting compared to untreated animals. Conversely, given their longer life expectancy, the natural loss of glomerular filtration during a year in humans and dogs would be limited, leaving enough functional nephrons to ensure kidney function after radiation-induced kidney damage.

Finally, a 3.7 MBq injected activity seems safe whereas the toxicity of 7.4 MBq needs to be recognised with regard to the toxicity of current cancer treatments and a risk/benefit balance. A clear toxicity to liver is observed at 11.1 MBq injected activity within 3–4 months after injection, indicating a clear toxicity at the corresponding dose to liver.

## Supporting Information

S1 Table Number of animals used in each experiment. (PDF)

S2 Table Renal and Hepatic toxicity. (PDF)

## Abbreviations

RIT: Radioimmunotherapy
BSA: Bovine Serum Albumin
ALT: Alanine aminotransferase
AST: Aspartate aminotransferase
BUN: Blood urea nitrogen
LET: Linear Energy Transfer
TRNT: Targeted radionuclide therapy
HES: Haematoxylin-Eosin-Saffron
PAS: Periodic Acid Schiff
MT: Masson’s Trichrome
WBC: White blood cells
RBC: Red blood cells
EMH: Extramedullary haematopoiesis
INCI: Intra-nuclear cytoplasmic invagination
RBE: Relative biological effectiveness
VOD: Veno-occlusive disease
