# Supplementary material for: Long-Term Toxicity of 213Bi-Labelled BSA in Mice
Source: PLoS One. 2016 Mar 16;11(3):e0151330. doi: 10.1371/journal.pone.0151330 (PMC4794211; doi:10.1371/journal.pone.0151330)
Supplement: S1 Table — (PDF) [file pone.0151330.s001.pdf]

Supplemental data 1: Number of animals used in each experiment

	Biodistribution and dosimetry	Weight and survival follow-up	Haematological toxicity evaluation	Hepatic and renal toxicity evaluation	Histology
^{125}I-BSA	n=12 (3 mice at 4 time points)				
^{213}Bi-BSA	n=12 (3 mice at 4 time points)	PBS, n=5<br>3.7 MBq, n=5<br>7.4 MBq, n=7<br>11.1 MBq, n=5	PBS, n=5<br>3.7 MBq, n=5<br>7.4 MBq, n=7<br>11.1 MBq, n=5	PBS, n=5<br>3.7 MBq, n=5<br>7.4 MBq, n=7<br>11.1 MBq, n=5	PBS, n=5<br>3.7 MBq, n=3<br>7.4 MBq, n=4<br>11.1 MBq, n=2
^{213}Bi-BSA					**26 W post injection:**<br>Control, n=3<br>11.1 MBq, n=4<br><br>**35 W post injection:**<br>Control, n=2<br>3.7 MBq, n=3<br>7.4 MBq, n=4
