# Supplementary material for: Long-Term Toxicity of 213Bi-Labelled BSA in Mice
Source: PLoS One. 2016 Mar 16;11(3):e0151330. doi: 10.1371/journal.pone.0151330 (PMC4794211; doi:10.1371/journal.pone.0151330)
Supplement: S2 Table — (PDF) [file pone.0151330.s002.pdf]

Supplemental data 2: Renal and Hepatic toxicity

Days	#	0	16	53	82	109	138	193	230	265	297	325	356	384
PBS	19													S
PBS	20													S
PBS	21													S
PBS	22													S
PBS	23													S
3.7 MBq	14													S
3.7 MBq	15													S
3.7 MBq	16							D						
3.7 MBq	17								D					
3.7 MBq	18													S
7.4 MBq	8													D
7.4 MBq	4									D				
7.4 MBq	11												D	
7.4 MBq	12										D			
7.4 MBq	7									D				
7.4 MBq	5											D		
7.4 MBq	6									D				
11.1MBq	2					D								
11.1MBq	3						D							
11.1MBq	9						D							
11.1MBq	10							D						
11.1MBq	13							D						

>2-fold increase in creatinine and BUN (azotaemia)

> 4-fold increase in AST and ALT

>2 fold increase in creatinine and BUN and > 4-fold increase in AST and ALT

S : Sacrificed at the end of the assay D : Dead during the course of the assay
